# Dynamic magnetic resonance of the wrist in psoriatic arthritis reveals imaging patterns similar to those of rheumatoid arthritis

**DOI:** 10.1186/ar1734

**Published:** 2005-04-01

**Authors:** Marco A Cimmino, Massimiliano Parodi, Stefania Innocenti, Giulia Succio, Simone Banderali, Enzo Silvestri, Giacomo Garlaschi

**Affiliations:** 1 Clinica Reumatologica, Dipartimento di Medicina Interna e Specialità Mediche, Università di Genova, Italy; 2 ESAOTE Biomedica, Genova, Italy; 3 Sezione di Diagnostica Radiologica, Dipartimento di Medicina Sperimentale, Università di Genova, Italy

## Abstract

This dynamic magnetic resonance imaging (MRI) study is concerned with a prospective evaluation of wrist synovitis in patients with psoriatic arthritis (PsA) in comparison with patients with rheumatoid arthritis (RA) and healthy controls. Fifteen consecutive patients with PsA, 49 consecutive patients with RA, 30 RA patients matched for disease severity with those with PsA, and 8 healthy controls were studied. MRI was performed with a low-field (0.2T), extremity-dedicated machine. After an intravenous bolus injection of gadolinium-diethylenetriaminepentaacetic acid, 20 consecutive fast spin-echo axial images of the wrist were obtained every 18 s. The enhancement ratio was calculated both as rate of early enhancement (REE), which shows the slope of the curve of contrast uptake per second during the first 55 s, and as relative enhancement (RE), which indicates the steady state of enhancement. The REE was 1.0 ± 0.6 in patients with PsA, 1.6 ± 0.7 in consecutive patients with RA, and 0.1 ± 0.1 in controls (*p *<*0.001*). The RE was 87.1 ± 39.2 in patients with PsA, 125.8 ± 48.0 in consecutive RA patients, and 15.5 ± 19.2 in controls (*p *<*0.001*). However, the same figures in matched RA patients were 1.3 ± 0.7 and 107.3 ± 48.2, respectively (not significant in comparison with PsA). Rheumatoid-like PsA and oligoarticular PsA did not differ from each other in terms of synovial enhancement. Dynamic MRI shows the same pattern of synovitis in patients with PsA and RA when the two groups are matched for disease severity. This technique cannot be used to differentiate PsA from RA. However, REE and RE were significantly higher in PsA than in normal controls, with only one instance of overlap between values found for the two groups.

## Introduction

Psoriatic arthritis (PsA), defined as the occurrence of seronegative arthritis and psoriasis, is a debated entity. The reasons for this uncertainty include disparity in the subgroups identified in the original description [1], lack of a validated case definition of PsA, possible inclusion of patients with enthesitis but without clear arthritis, the elusive link between skin and joint diseases, and similarities with rheumatoid arthritis (RA). It is not known if PsA and RA are completely distinct diseases, or if PsA is a form of RA modified by the coexisting psoriasis. This second hypothesis is brought into doubt by the original observation that articular involvement is chiefly symmetric and polyarticular in RA, but oligoarticular and asymmetric in PsA.

However, subsequent studies have shown that the most common subset of PsA is a symmetric polyarthritis resembling RA [2]. In particular, oligoarticular disease may be a characteristic of PsA mainly at presentation [3], and symmetry is a function of the number of joints involved but not of the type of arthritis [4]. On average, however, PsA is characterized by a milder degree of synovitis than RA, with only 8% of patients developing erosions of the hand. Therefore, therapy with disease-modifying anti-rheumatic drugs is rarely needed [5].

Magnetic resonance imaging (MRI) has helped in the evaluation of PsA by suggesting that the primary site of inflammation is extrasynovial and that synovial inflammation may be a secondary phenomenon [6]. In addition to morphologic studies of the joint, MRI may be used also to evaluate synovial membrane inflammation through a dynamic, contrast-enhanced technique. By this method, we evaluated gadolinium perfusion of the synovial membrane to differentiate active RA from RA in remission [7]. In the present paper, the same technique has been used to study patients with PsA. The main goals of the study were to investigate whether synovitis differs between PsA and RA, and, if so, whether the difference is intrinsic to the type of arthritis or is due to the severity and duration of the disease.

## Materials and methods

### Patients

Fifteen consecutive patients with PsA, defined as the simultaneous occurrence of active arthritis and psoriasis, were prospectively studied. Eight patients had a polyarticular rheumatoid-like pattern of arthritis and seven had monoarthritis or oligoarthritis, associated in one patient with axial involvement. Dynamic MRI data for these patients were compared with those of 49 consecutive patients with active RA (group I), diagnosed according to the criteria of the American Rheumatism Association [8]. An additional group of 30 patients with RA, matched with those with PsA in terms of age, disease duration, and number of involved joints, was considered (group II). These patients were identified in ongoing follow-up studies. Because of difficulties in matching, nine of these patients were recruited from groupI RA patients. All the patients considered in this study were seen at the Rheumatological Clinic of the University of Genoa, Italy, either in the clinical ward or as outpatients, and had clinical inflammatory involvement of at least one wrist. Clinical parameters, such as duration of arthritis, early-morning stiffness, fatigue, number of tender and swollen joints, and type of treatment, were evaluated before MRI. At the same time, blood was drawn for the determination of the erythrocyte sedimentation rate (Westergren method), C-reactive protein, and IgM rheumatoid factor by standard laboratory methods. In addition, a control group of eight healthy volunteers, who did not report any history of joint disease and were negative for signs of arthritis on clinical examination, was also studied. All subjects gave their informed consent to the protocol, which had been approved by the ethics committee of the Department of Internal Medicine of the University of Genoa. Demographic and clinical characteristics of the three groups of patients and the group of controls are reported in Table 1.

### Methods

MRI of the wrist was performed with a low-field (0.2T), extremity-dedicated machine (Artoscan™, Esaote, Genoa, Italy) equipped with a permanent magnet and with a dedicated hand-and-wrist coil 13 cm in diameter, as previously described [6]. The hand was fixed in neutral position and the fingers in extended position with the thumb up, by the application of several cushions. The field of view was 120 mm and allowed the evaluation of the carpal bones, the metacarpal bases, and the distal radius and ulna. Slice thickness was 5 mm and the interslice gap was 0.3 mm. The sequence used was a spin echo (TR/TE = 100/16 ms, matrix = 160 × 128, FOV = 150 × 150), which was acquired in the axial plane. In patients with arthritis, the more severely affected wrist was examined. In patients with equal involvement of the wrists, and in normal controls, the right wrist was examined. Patients and controls were instructed to avoid intense activity involving the wrists in the 24 hours preceding the examination.

**Figure 1:**
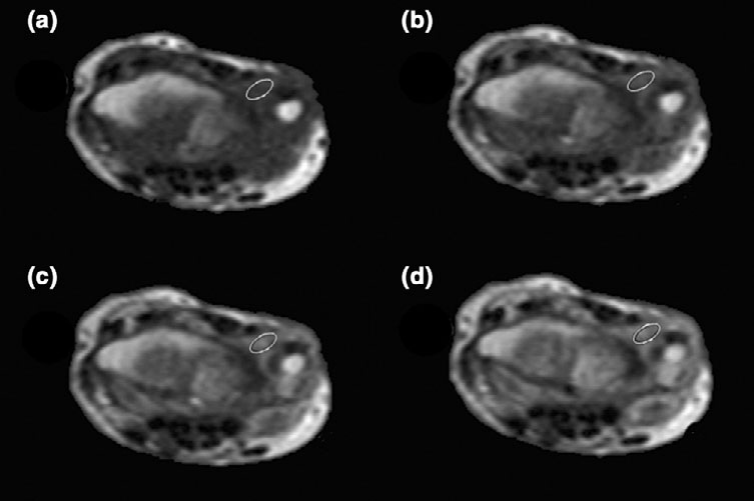
Dynamic gadolinium-diethylenetriamine pentaacetic acid (Gd-DTPA)-enhanced MRI of the wrist in a patient with psoriatic arthritis. Sequence **(a) **shows the precontrast image; sequences **(b–d) **show images acquired after 36, 90, and 180 s, respectively. The region of interest on which the enhancement curve has been calculated is outlined. Gd-DTPA, dynamic gadolinium-diethylenetriamine pentaacetic acid; MRI, magnetic resonance image.

After the wrist was positioned in the gantry, the first image was acquired. Then an intravenous bolus injection of 0.2 ml/kg of Gd-DTPA (gadolinium-diethylenetriaminepentaacetic acid) (Omniscan, Schering, Germany) was given manually in 30 s through a 21-mm butterfly needle into a cubital vein. Twenty consecutive fast images of three slices of the wrist, the first of which was positioned tangential to the radius, were repeated every 18 s thereafter. The rate of enhancement was evaluated by a radiologist on the slice that showed the highest visual enhancement. It was calculated as Δ on a small, elliptical region of interest (ROI) of synovial membrane of approximately 25 mm^2 ^positioned in the area of highest visual enhancement (Fig. 1). Entheses and synovial sheaths were not included in the ROI. In healthy controls, the synovial membrane was more difficult to identify. In these cases, the area where the synovium was thought to be located was chosen on the basis of anatomic landmarks and comparison of pre- and post-enhancement images. For this reason, the elliptical ROI was usually smaller in controls than in patients. This corresponded usually to the median and dorsal area of the wrist.

The images were processed blind to the clinical and laboratory findings. The enhancement ratio was calculated both as rate of early enhancement (REE) per second during the first 55 s according to the formula

REE_55 _= (S_55 _- S_0_)/(S_0 _× 55) × 100%

and as relative enhancement (RE) at t seconds according to the formula

RE_t _= (S_t _- S_0_)/S_0 _× 100%

where S_0 _and S_t _are the signal-to-noise ratios, before and t seconds after contrast injection, calculated as ratio between the signal measured in the ROI and the standard deviation of the background noise. The study of enhancement after 55 s was chosen because it showed maximal enhancement difference between knees with clinically inactive or active disease in a previous study [9]. In addition, the signal was normalized to the bone to reduce noise. The REE shows the slope of the curve of contrast uptake tangential to the α angle and is steeper if inflammation is higher. The RE indicates the steady state of enhancement. The intra- and inter-observer mean percentage variations for REE were 3.9% (range 0.5% to 14.3%) and 2.8% (range 0% to 5.1%), respectively, in 18 wrists. Intra- and inter observer mean percentage variation for RE were 1.9% (range 0% to 9.3%) and 1.9% (range 0.05% to 6.4%) (manuscript in preparation).

### Statistical evaluation

Means were compared by the Student's *t*-test or by one-way analysis of variance (ANOVA) if their distribution was normal and by the Wilcoxon test with Mann–Whitney correction or Kruskal–Wallis ANOVA when the distribution was nonparametric. Frequencies were compared using the Fisher exact test. Correlations were calculated by the Pearson or Spearman rank tests. *P *values less than 0.05 were considered significant.

## Results

### Comparison between PsA and consecutive RA patients

**Figure 2:**
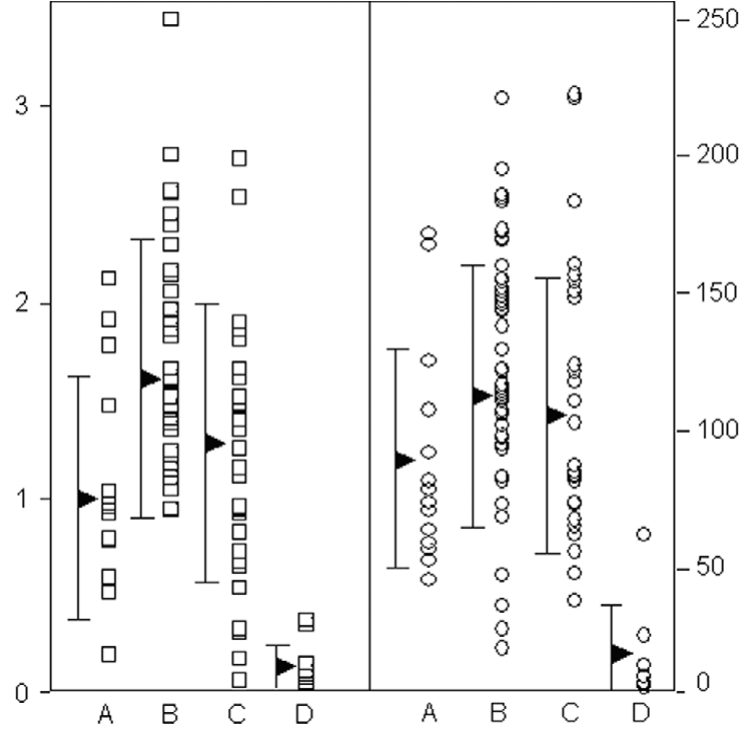
Individual values of REE (left) and RE (right) in patients with arthritis and controls. (A) Patients with psoriatic arthritis, (B) group-I rheumatoid arthritis, or (C) groupII rheumatoid arthritis; (D) controls. Triangles indicate the mean values. Vertical bars indicate standard deviations. RE, relative enhancement; REE, rate of early enhancement.

The demographic and clinical data for patients and controls are reported in Table 1 in comparison with RA patients of group I. PsA patients had a less skewed female-to-male ratio, had a lower tender joint count, and were less frequently positive for IgM rheumatoid factor. All the other clinical characteristics were similar in the two groups. Controls were younger than the patients of the other two groups. Dynamic MRI was performed in all subjects without causing any discomfort or adverse events. The mean duration of the complete examination was 15 min. REE and RE were significantly different in the three groups (*p *<*0.001*) (Table 1; Fig. 2). The values were highest for the group with RA, followed by that with PsA and controls. In PsA and RA patients, the REE ranged from 0.14% to 2.13% per second, and from 0.08% to 3.49% per second, respectively. In controls, it ranged from -0.02% to 0.37% per second.

Nonsteroidal anti-inflammatory drugs were being used by 14 (93.3%) of 15 PsA patients, and by 40 (81.6%) of 49 RA patients (ns). Sulphasalazine was used in 7 (47.7%) of 15 PsA patients and in 17 (34.7%) of 49 RA patients (ns). Conversely, patients with PsA were treated less frequently with prednisone (4 of 15, or 26.7%, vs 36 of 49, or 73.5%; *P *= 0.002) or methotrexate (2 of 15, or 13.3%, vs 21 of 49, or 42.9%, *P *= 0.06) than patients with RA. Dosages were similar in the two groups of patients (data not shown).

### Comparison between PsA and matched RA patients

To exclude the possibility that a lower disease activity in PsA patients could account for the observed dynamic MRI difference, 2 RA patients were matched for age and number of tender and swollen joints to each of the 15 patients with PsA. The clinical and laboratory characteristics of the PsA patients and of the new RA control group (group  II) were similar, with no statistically significant difference. Only positivity for IgM rheumatoid factor was higher in RA patients (*P *< 0.05) (Table 1).

Treatment in the matched RA group (II) was similar to that seen in the groupII RA patients. The use of nonsteroidal anti-inflammatory drugs and sulphasalazine was not different in PsA patients from that in matched RA patients. Patients with PsA were treated less frequently with prednisone (26.7% vs 70%; *P *= 0.01) and with methotrexate (13.3% vs 46.6%, *P *= 0.046) than patients with RA, but dosages were similar.

**Figure 3:**
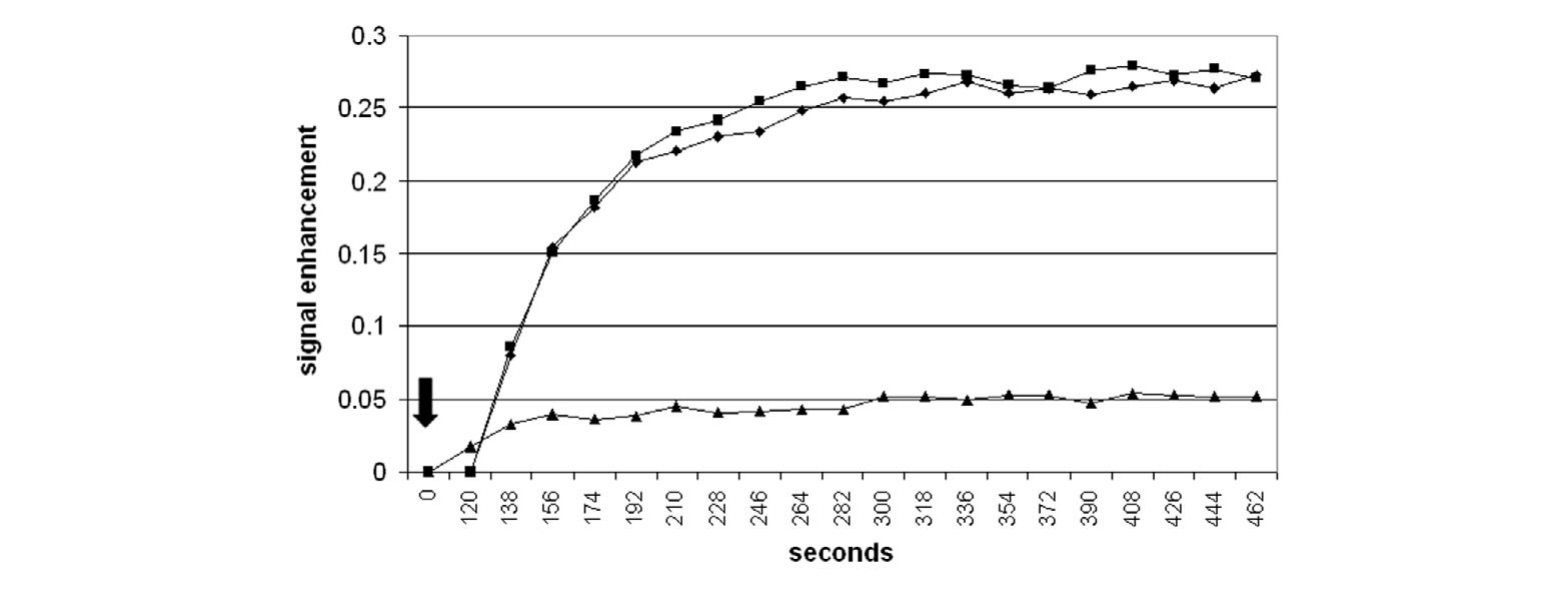
Slope of the mean enhancement curves in patients with arthritis and in controls. Patients with psoriatic arthritis (squares) or with rheumatoid arthritis (diamonds) matched for demographic characteristics and disease severity; controls (triangles). The arrow indicates the time of gadolinium-diethylenetriamine pentaacetic acid infusion.

REE and RE were not different between PsA and RA of similar severity (Table 1; Fig. 2). Figure 3 compares the mean values of the curves in the three subgroups, that is, patients with PsA, groupII RA patients, and healthy controls. Before Gd-DTPA infusion and for the first 36 s after infusion, the three curves were almost identical. However, a highly significant difference in enhancement was seen by ANOVA at all the following time points (*P *= 0.003 at t = 156 s, *p *<*0.001 *at t = 174 s, and *P *< 0.001 thereafter). The curves identified two groups of patients, one being patients with PsA or groupII RA and the other being controls.

### Correlations between dynamic MRI and clinical and laboratory findings

REE was 0.8 ± 0.5 in patients with rheumatoid-like PsA and 1.2 ± 0.6 in those with monoarthritis or oligoarthritis (ns). Values for RE were 78 ± 28.2 and 97.5 ± 49.2, respectively. This difference was not significant. REE and RE were not correlated with clinical and laboratory findings in PsA. There was a tendency to an association between REE and number of swollen joints in the RA patients of group II (*P *= 0.07).

## Discussion

Dynamic MRI is a promising method for the investigation of patients with arthritis. In our previous experience, it could differentiate patients with active RA from those in remission and from controls [7]. In another study of arthritic knees, dynamic gadolinium-enhanced MRI showed increased contrast diffusion in comparison with controls [9]. These results are in keeping with the well-known ability of MRI to detect synovitis in diseased joints [10]. We therefore decided to evaluate patients with PsA and to compare them with RA, in view of the existing debate on similarities and differences between the two diseases.

After intravenous injection, Gd-DTPA, a relatively small molecule, rapidly diffuses to highly vascularized tissues, such as the inflamed synovial membrane, and the rapidity and amount of diffusion seem to be related to the number, size, and permeability of synovial vessels as well as to the volume of the synovial membrane [11]. When PsA patients were compared with an unselected group of consecutive RA patients, the REE and RE were significantly lower in those with PsA. This observation could lead to the interpretation that inflammation of the synovial membrane is lower in PsA, a finding supported by several epidemiological and clinical studies [5]. However, the suspicion arose that the two groups of patients might not be comparable, because of higher disease activity in patients with RA. In fact, the number of tender joints was significantly higher in RA patients (Table 1), although the other disease characteristics were only slightly higher. We had the opportunity to match each patient with PsA with two RA patients for number of tender and swollen joints. As a result, a new RA group (group II) was formed that included some of the consecutive RA patients and several new patients drawn from ongoing follow-up studies. The two groups of patients were clinically fully comparable. Of course, the percentage of patients with IgM rheumatoid factor could not be easily matched in this type of study. Another difference between groups included the more frequent administration of prednisone and methotrexate in RA patients. In fact, matching also for treatment was not possible due to the relatively small number of RA patients. It could be argued that prednisone and methotrexate could affect dynamic MRI per se by directly acting on neovascularization. However, these two drugs were used more frequently in both RA groups, which behave differently from PsA as far as REE and RE are concerned. It is therefore unlikely that their direct effect on dynamic MRI could have influenced our results, which are, rather, explained by a difference in inflammation.

After matching, the REE and RE were not significantly different in the two groups of patients (Fig. 3). This finding indicates that, at comparable levels of disease severity, synovitis revealed by dynamic MRI presents a pattern in PsA that is similar to that of RA. This finding contradicts the common belief that PsA, on the whole, is a mild form of arthritis. The amount of contrast agent transported to the inflamed synovial membrane is probably a result of the number, size, and permeability of vessels and volume of the synovial membrane itself. A greater number of synovial vessels per squaremillimetre of tissue has been demonstrated in PsA than in RA [12]. Conversely, significantly less lining-layer hyperplasia was demonstrated in PsA in the same study [12]. The net effect of these two contrasting features on Gd-DTPA diffusion is not known and could be assessed only by comparing dynamic MRI and synovial membrane histology in the same joints. Other vessel characteristics of PsA synovial membrane that could play a role in contrast agent diffusion are the marked thickening of the vessel wall [13] and the peculiar, tortuous vascular pattern [14].

Dynamic MRI highlights the similarity of the synovial membrane in PsA and RA and supports the view that the two conditions may be more similar than is usually believed, at least as far as disease activity is concerned. This observation is in keeping with the fact that the same types of treatment, including sulphasalazine, methotrexate, leflunomide, and anti-tumor-necrosis-factor-α compounds, are effective in RA and PsA. As a result, dynamic MRI cannot be used to differentiate the two diseases. However, both REE and RE data were significantly higher in PsA than in healthy controls, with only one case of overlap between the two conditions.

A more efficient way of differentiating PsA from RA by MRI is to study the pattern of joint involvement. Jevtic and colleagues [15] showed that inflammation is localized within the joint capsule in the small joints of the hand of RA patients, whereas PsA patients also show extracapsular involvement, with thickened collateral ligaments and oedema of the neighbouring soft tissues. In another study of the knee, focal perientheseal high signal outside the joint, and bone marrow oedema at entheseal insertions were typical features of patients with spondyloarthropathy [16]. Our study, in which we aimed to compare the degree of synovitis in the two forms of arthritis, did not take into consideration damage to entheses and synovial sheaths, areas that could be more effective in the differential diagnosis and deserve a separate investigation.

Our results were obtained with a low-field extremity-dedicated MRI device. The lack of discrimination between RA and PsA with a 0.2-T MRI device could be overcome with other MRI protocols, such as different pulse sequences, field strengths, or magnet types. High-field MRI machines have a better signal-to-noise ratio and could be hypothetically more sensible in the evaluation of enhancement. Evaluation of the sensitivity of dynamic MRI is still in its early stages. Within RA, we showed that this technique can discriminate between different degrees of clinical activity [7]. These considerations suggest that differences in dynamic MRI between RA and PsA, if present, should be relatively small. There are no papers directly comparing dynamic MRI obtained with low- and high-field machines. Results of another recent study by Palosaari and colleagues on wrist RA [17], made using a low-field MRI, are difficult to compare with ours, because technical features such as type of sequence, imaging parameters, acquisition plane, number of sequences, and amount of contrast agent were different. In addition, the cohort of RA patients in that study, being affected by early disease, was different. The absolute values of signal enhancement were higher in Palosaari's study. By contrast, a third study [18] on the rheumatoid knee performed with a 1.5-T unit showed absolute enhancement values similar to those that we obtained.

The intraobserver/interobserver agreement in evaluating dynamic enhancement was very high. This may be surprising, in view of the fact that the examination process included selection of slice, of maximal enhancing area, and of the size of the ROI. We feel that high reproducibility may have been facilitated by a significant association between enhancement figures of elliptical areas in the three sequentially acquired wrist slices [unpublished observations in [7]]. This makes the choice of the slice less important. In addition, the area of maximum enhancement, exclusive of entheses and tendon sheaths, is very often located on the dorsal side of the wrist and is relatively small, another constraint of choice for the examiner. Elliptical ROIs, although apparently less logical from a pathophysiological point of view than ROIs outlining the enhanced synovial membrane, were chosen to improve reproducibility and standardization. A recent paper on contrast-enhanced dynamic MRI of coronal slices of the wrist also showed high intraobserver reliability [17].

Only one PsA patient was positive for rheumatoid factor. She did not show a rheumatoid-like pattern of joint involvement, but had monoarthritis of the right wrist and dactylitis, which is not typical of RA. Of the 15 PsA patients, 8 had a rheumatoid-like pattern of arthritis and 7 had oligomonoarthritis. Dynamic MRI was not significantly different between these two groups, reinforcing the suggestion that severity of the arthritis, and not its type or subtype, is associated with MRI findings.

The patients were selected on the basis of clinical involvement of the wrist. This prerequisite was set because, if all consecutive patients had been enrolled, many more RA patients than PsA patients would have had wrist involvement, thus making the two groups more difficult to compare. In a previous study by our team [7], RA patients in remission and without wrist arthritis had dynamic MRI values significantly lower than those with active disease and wrist involvement. We do not know if the results obtained in unaffected wrists of otherwise active arthritis patients reflect local (wrist) or general disease activity. No correlation was found between indexes of severity and dynamic MRI in either PsA and RA. This last finding is surprising in view of our previous results on the high correlation between clinical and laboratory indexes of RA inflammation and dynamic MRI. However, the inclusion of severely active arthritis only – with exclusion of patients in remission – and the relatively low mean number of affected joints in the patients with PsA and in the matched RA controls may account for the difference. Nonetheless, a tendency to correlation between number of swollen joints and REE, which in our experience is the more sensitive measure, was observed.

## Conclusion

We have shown that dynamic MRI gives similar results in PsA and RA, suggesting that the type and degree of inflammatory process is similar in the two diseases.

## Abbreviations

ANOVA = analysis of variance; Gd-DTPA = gadolinium-diethylenetriaminepentaacetic acid; MRI = magnetic resonance imaging; ns = not significant; PsA = psoriatic arthritis; RA = rheumatoid arthritis; RE = relative enhancement; REE = rate of early enhancement; ROI = region of interest.

## Competing interests

SI is an employee of ESAOTE, the manufacturer of the magnetic resonance device.

## Authors' contributions

MAC and MP contributed to the conception and design of the study, to the clinical and MRI evaluation of the patients, to the analysis and interpretation of data, to the drafting of the article, and to the critical revision of the article for important intellectual content; SI contributed to the analysis and interpretation of data and to the critical revision of the article for important intellectual content; ES and GG contributed to the conception and design of the study, to the analysis and interpretation of data, and to the critical revision of the article for important intellectual content; GS and SB contributed to the conception and design of the study, to the clinical and MRI evaluation of the patients, to the analysis and interpretation of data, and to the critical revision of the article for important intellectual content. All authors read and approved the final manuscript.

## Figures and Tables

**Table 1:** Demographic, clinical, and laboratory characteristics of patients with psoriatic arthritis, rheumatoid arthritis, and controls

Characteristic	Psoriatic arthritis	Rheumatoid arthritis (group I)	Matched rheumatoid arthritis (group II)	Controls
Number of patients	15	49	30	8
Age (years)	55.7 ± 10.7	57.6 ± 14.6	56.3 ± 16.3	38.1 ± 21.9
Sex (women/men)*	8/7	42/7	20/10	4/4
Disease duration (months)	63.5 ± 62.7	93.5 ± 98.7	78.2 ± 86.2	NA
Morning stiffness (minutes)	70.4 ± 55.7	96.2 ± 96.3	76.8 ± 92.1	NA
Number of tender joints*	8.0 ± 5.5	13.7 ± 9.4	9.1 ± 7.3	0
Number of swollen joints	5.3 ± 4.4	8.0 ± 5.8	5.0 ± 3.9	0
Ritchie index	8.3 ± 5.7	11.6 ± 6.9	7.0 ± 5.1	0
ESR (mm/h)	34.7 ± 21.8	49.1 ± 31.6	45.5 ± 35.7	ND
CRP (mg/l)	30.4 ± 39.4	30.3 ± 34.4	26.6 ± 36.1	ND
Number of patients with IgM rheumatoid factor**	1 (6.7%)	33 (67.3%)	18 (60%)	ND
Rate of early enhancement^§^	1.0 ± 0.6	1.6 ± 0.7	1.3 ± 0.7	0.1 ± 0.1
Relative enhancement ^§^	87.1 ± 39.2	125.8 ± 48.0	107.3 ± 48.2	15.5 ± 19.2

Values are expressed as means ± standard deviations except for sex and IgM rheumatoid factor, for which the percentage of positive patients is reported. *P < 0.05 for the comparison between PsA and groupI RA; **P < 0.001 for the comparison between PsA and groupI RA and *P *< 0.05 for the comparison between PsA and groupII RA; ^§^*P *< 0.001 by one-way analysis of variance between PsA patients, groupI RA patients, and controls.
